# Reference‐based multiple imputation for missing data sensitivity analyses in trial‐based cost‐effectiveness analysis

**DOI:** 10.1002/hec.3963

**Published:** 2019-12-17

**Authors:** Baptiste Leurent, Manuel Gomes, Suzie Cro, Nicola Wiles, James R. Carpenter

**Affiliations:** ^1^ Department of Medical Statistics London School of Hygiene and Tropical Medicine London UK; ^2^ Department of Applied Health Research University College London London UK; ^3^ Imperial Clinical Trials Unit, School of Public Health Imperial College London London UK; ^4^ Population Health Sciences, Bristol Medical School University of Bristol Bristol UK; ^5^ MRC Clinical Trials Unit University College London London UK

**Keywords:** controlled imputation, cost‐effectiveness analysis, missing data, missing not at random, multiple imputation, randomised trial, reference‐based, sensitivity analysis

## Abstract

Missing data are a common issue in cost‐effectiveness analysis (CEA) alongside randomised trials and are often addressed assuming the data are ‘missing at random’. However, this assumption is often questionable, and sensitivity analyses are required to assess the implications of departures from missing at random. Reference‐based multiple imputation provides an attractive approach for conducting such sensitivity analyses, because missing data assumptions are framed in an intuitive way by making reference to other trial arms. For example, a plausible not at random mechanism in a placebo‐controlled trial would be to assume that participants in the experimental arm who dropped out stop taking their treatment and have similar outcomes to those in the placebo arm.

Drawing on the increasing use of this approach in other areas, this paper aims to extend and illustrate the reference‐based multiple imputation approach in CEA. It introduces the principles of reference‐based imputation and proposes an extension to the CEA context. The method is illustrated in the CEA of the CoBalT trial evaluating cognitive behavioural therapy for treatment‐resistant depression. Stata code is provided. We find that reference‐based multiple imputation provides a relevant and accessible framework for assessing the robustness of CEA conclusions to different missing data assumptions.

## 1 INTRODUCTION

Cost‐effectiveness analysis (CEA) of randomised trials provides an important source of information for decision making but is often limited by incomplete data collection. For example, participants may withdraw before the end of the study or fail to complete a questionnaire. This is particularly common in longitudinal studies, where data are collected at multiple follow‐up points, as is often the case in CEA. There has been substantial progress in methods for handling missing data in CEA particularly those that allow valid inferences under the assumption that data are missing at random (MAR; Briggs, Clark, Wolstenholme, & Clarke, 2003; Burton, Billingham, & Bryan, 2007; Faria, Gomes, Epstein, & White, 2014; Lin, 2003; Manca & Palmer, 2005; Oostenbrink & Al, 2005; Willan, Lin, & Manca, 2005). In recent years, there has been an increase in the uptake of methods such as multiple imputation (Gabrio, Mason, & Baio, 2017; Leurent, Gomes, & Carpenter, 2018; Noble, Hollingworth, & Tilling, 2012). The MAR assumption often provides a desirable starting point for missing data analyses as it implies that any differences between individuals with missing and complete information can be explained by differences in the observed data. However, this assumption may not always hold, as the missingness could depend on unobserved values, that is, data are missing not at random (MNAR; Little & Rubin, 2002). For example, participants in poorer health may be less likely to complete health‐related quality of life (QoL) questionnaires (e.g., EQ‐5D; EuroQol Group, 1990), conditional on their observed characteristics (Faria et al., 2014; Leurent et al., 2018).

Because the true missing data mechanism is unknown, methodological guidelines recommend conducting sensitivity analyses to departures from the MAR assumption, considering alternative, plausible MNAR mechanisms (Burzykowski et al., 2010; Committee for Medicinal Products for Human Use [CHMP], 2010; Faria et al., 2014; Little et al., 2012). However, these sensitivity analyses are not routinely conducted (Bell, Fiero, Horton, & Hsu, 2014; Gabrio et al., 2017; Leurent, Gomes, & Carpenter, 2018), perhaps due to the lack of accessible methods or because of the challenges of formulating relevant missing data assumptions beyond MAR. One approach that is receiving increasing attention in clinical trials is reference‐based multiple imputation (Carpenter, Roger, & Kenward, 2013; Keene, Roger, Hartley, & Kenward, 2014; Kenward, 2015; Little & Yau, 1996). This approach recognises that individuals with missing data could differ from those who complete the study, and—reflecting this—the data are imputed using a different distribution. For example, in a placebo‐controlled drug trial, participants in the experimental arm who dropout may stop taking their treatment and be expected to have similar outcomes to those in the placebo arm. A key advantage of this approach over other methods that have been proposed (Gabrio, Daniels, & Baio, 2018; Gabrio, Mason, & Baio, 2019; Leurent, Gomes, Faria, et al., 2018; Mason, Gomes, Grieve, & Carpenter, 2018) is that the departure from MAR is captured in a qualitative way, making the formulation of the problem more intuitive and accessible to a broader audience, including clinicians and decision makers.

Drawing on recent work (Carpenter et al., 2013), this paper extends and illustrates the reference‐based multiple imputation approach to address MNAR data in trial‐based CEA. In particular, we focus on adapting the approach to jointly model costs and effectiveness and allow for different patterns of missingness on cost and effectiveness endpoints over time.

This paper is organised as follows: Section 2 introduces the CoBalT trial, which is used as a motivating example to illustrate the methods. Section 3 introduces the reference‐based multiple imputation approach, its extension to the CEA framework, and its implementation in Stata (StataCorp, 2017). Section 4 illustrates the methods applied to the CoBalT trial. The paper finishes with a discussion of the proposed methods.

## 2 CASE STUDY

### 2.1 Overview of the CoBalT trial

CoBalT was a two‐arm individually randomised controlled trial of cognitive behavioural therapy (CBT) as an adjunct to pharmacotherapy for treatment‐resistant depression (Wiles et al., 2014, 2013). Patients with treatment‐resistant depression were recruited from U.K. primary care practices between 2008 and 2010 and randomised to either usual care for depression (including pharmacotherapy) or to CBT in addition to usual care. CBT consisted of 12 to 18 sessions delivered by a trained therapist at the general practice or a nearby location and followed standard CBT manuals (Thomas et al., 2012). The trial's primary outcome was clinical response, defined as a 50% reduction in depressive symptoms (Beck Depression Inventory‐II; Beck, Steer, & Brown, 1996) at 6 months compared with baseline.

### 2.2 Cost‐effectiveness analysis

A 1‐year CEA was conducted alongside the trial to assess the cost‐effectiveness of CBT in addition to usual care and has been reported in detail elsewhere (Hollinghurst et al., 2010; Wiles et al., 2014). For the purpose of this article, we follow broadly the CEA methods described in Hollinghurst et al. (2010) with some simplifications made to allow a clearer focus on the relevant methodology (e.g., focussing only on unadjusted cost‐utility analysis and costs from the National Health Service and Personal Social Service perspective). Briefly, health‐related QoL was measured by the EQ‐5D‐3L (EuroQol Group, 1990) at baseline, 6, and 12 months and converted into utility scores using a standard set of U.K. valuations (Dolan, 1997). Quality‐adjusted life‐years (QALYs) were derived by the “area under the curve” approach, combining both time and utility scores (Drummond, Sculpher, Claxton, Stoddart, & Torrance, 2015). Costs were measured from the National Health Service and Personal Social Service perspective over 12 months. Primary care use was derived from the general practice records at the end of the trial, and additional health and social service use were measured by patient questionnaires at 6 and 12 months. Costs were not collected at baseline. For the purposes of this article, we focused on imputing the total cost variable. Missing QoL data and total costs were imputed under different assumptions using the referenced‐based multiple imputation approach described in Section 3. Participants' baseline QoL, age, sex, and Beck Depression Inventory‐II were used as covariates in the imputation model, and a set of 100 imputations were performed. The imputed QALYs were then derived by the area under the curve approach based on the imputed QoL at baseline, 6, and 12 months. The resulting multiply‐imputed datasets were analysed using Rubin's rules (Rubin, 1987). Mean differences between arms in QALYs and costs (and 95% confidence intervals) were estimated using unadjusted linear regression and divided to obtain the incremental cost‐effectiveness ratio (ICER) of CBT compared with usual care. The probability of CBT being cost‐effective at different willingness to pay thresholds (and the resulting cost‐effectiveness acceptability curve; Fenwick, O'Brien, & Briggs, 2004) were derived using unadjusted “seemingly unrelated regressions” (Willan, Briggs, & Hoch, 2004) for QALYs and for costs. All analyses were performed in Stata version 15 (StataCorp, 2017).

### 2.3 Missing data patterns and descriptive results

The trial enrolled 469 participants, and 101 (21.5%) had some cost or QoL data missing. Table 1 shows the frequency of each missing data pattern for the cost and QoL variables. The cost endpoint had slightly more missing data than the QoL endpoint, and the missing data were mostly monotone (when QoL was unobserved at 6 months, QoL at 12 months and total costs tended to be missing as well), but there were also some participants with interim‐missing data (QoL missing at 6 months but observed at 12 months). The majority of participants were in the “completers” pattern (78.5%), and each of the missing data pattern had a small number of participants (from 0 to 23 per arm), with the most common being not having any follow‐up data (9%). There was no important difference between arms, with 77.4% of participants providing complete data in the usual care arm and 79.5% in the CBT arm. Missing data were mostly due to participants withdrawing from the study or being lost to follow‐up during the trial and were more common in men and younger participants (Wiles et al., 2014).

**Table 1:** Missing data patterns of CoBalT quality of life and cost variables

Missing data pattern				Usual care (*N* = 235)		CBT (*N* = 234)		Total (*N* = 469)	
QoL baseline	QoL 6 months	QoL 12 months	Total cost	*n*	%	*n*	%	*n*	%
✓	✓	✓	✓	182	77.4	186	79.5	368	78.5
✓	✓	✓	✘	13	5.5	6	2.6	19	4.1
✓	✘	✓	✓	0	0.0	2	0.9	2	0.4
✓	✓	✘	✘	18	7.7	14	6.0	32	6.8
✓	✘	✓	✘	3	1.3	3	1.3	6	1.3
✓	✘	✘	✘	19	8.1	23	9.8	42	9.0

*Note*. Ticks indicate observed data; crosses indicate missing data.

Abbreviations: CBT, cognitive behavioural therapy; QoL, health‐related quality of life measured by the EQ‐5D‐3L.

**Figure 1:**
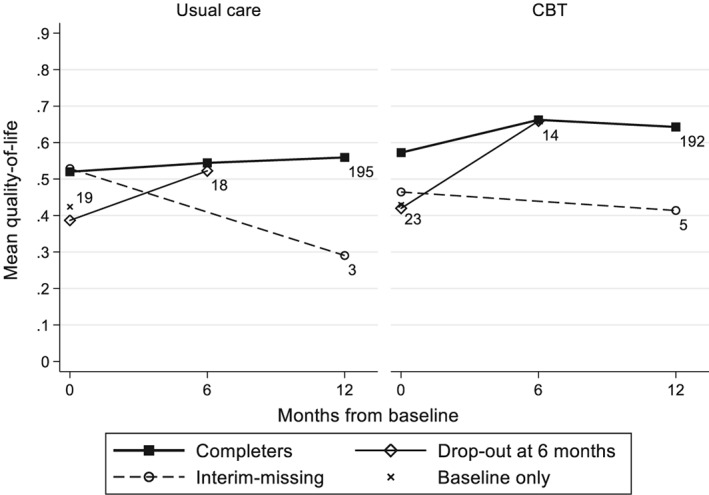
CoBalT mean quality of life scores, by treatment arm and missing data pattern. The number of participants in each pattern is indicated next to the last observation. Linear change is assumed between time points. CBT, cognitive behavioural therapy

Table 2 reports the observed mean and standard deviation for the cost and QoL variables. The mean QoL over time is also shown by missing data pattern in Figure 1. In the participants with complete QALY data, the QoL tended to increase over time, with a greater improvement in the CBT arm than the usual care arm, particularly between baseline and 6 months. Participants with missing data tended to have a lower QoL at baseline.

**Table 2:** Summary statistics of CoBalT quality of life and cost variables

Variable	Usual care (*N* = 235)			CBT (*N* = 234)		
	*n*	*M*	*SD*	*n*	*M*	*SD*
QoL baseline	235^a^	0.502	0.311	234	0.547	0.315
QoL 6 months	213	0.542	0.329	206	0.662	0.303
QoL 12 months	198	0.555	0.358	197	0.637	0.338
QALYs	195	0.542	0.292	192	0.635	0.279
Total cost (£)	182	799	725	188	1,803	1,115

Abbreviations: CBT, cognitive behavioural therapy; M, mean; QALYs, quality‐adjusted life‐years; QoL, health‐related quality of life measured by EQ‐5D‐3L; SD, standard deviation.

a One missing baseline QoL was mean‐imputed.

The primary CEA (Hollinghurst et al., 2010) was conducted using multiple imputation, assuming missing data were MAR. However, due to the nature of the illness, it was argued that those with poorer outcomes could have been more likely to dropout of the trial (i.e., data may be MNAR). To ensure that the study provides sound evidence, it is therefore important to assess the extent to which the cost‐effectiveness inferences are robust to departures from the primary MAR assumption. Reference‐based imputation provides a particularly appealing framework to conduct these sensitivity analyses under varying missing data assumptions, as we will see in the following sections.

## 3 REFERENCE‐BASED MULTIPLE IMPUTATION

This section starts by introducing the basic principles of the reference‐based multiple imputation approach drawing on recent work (Carpenter et al., 2013). We then provide some technical details and describe how the approach can be extended for the CEA setting and implemented in standard statistical software.

### 3.1 Overview

Reference‐based multiple imputation is part of the reference‐based (or “controlled” or “placebo‐based”) approaches to handling missing data (Ayele, Lipkovich, Molenberghs, & Mallinckrodt, 2014; Carpenter et al., 2013; Keene et al., 2014; Kenward, 2015; Little & Yau, 1996; Lu, 2014; Tang, 2018), which belong to a broader class of “pattern mixture models,” modelling MNAR data by allowing for a different data distribution by pattern of missingness (Little, 1993; Ratitch, O'Kelly, & Tosiello, 2013). Reference‐based multiple imputation can be seen as an extension of ad hoc single imputation MNAR methods, such as assuming “missing = still smoking” commonly used in smoking cessation trials (West, Hajek, Stead, & Stapleton, 2005) but appropriately capturing random variations and imputation uncertainty in a multiple imputation framework. Instead of a single imputation, an appropriate distribution is used to draw multiple plausible values. This distribution can come from any “reference” group, but a typical choice in randomised trials would be to use the control arm information. For example, in a placebo‐controlled trial, we may wish to use the distribution from the placebo arm to impute outcomes of active arm individuals who dropped out (assuming these have stopped taking their treatment). Multiple imputation provides a convenient framework to implement this approach, because it naturally builds on the MAR elements (Carpenter et al., 2013). Once a multivariate model has been fitted assuming MAR, the different elements of the model can be used as “building blocks” to construct the desired distribution under MNAR. We describe this more formally in the next section.

### 3.2 Generic algorithm

Consider a randomised controlled trial, where an outcome (say QoL) is measured at multiple time points. Let *i* = 1, … , *N* index the *N* participants randomised in the trial, and *T*_*i*_ indicate the randomisation arm of participant *i*. Let *j* index the time points, *j* = 0, … , *J*, with *j* = 0 the baseline measurement. *Y*_*ij*_ denotes the value of the outcome for participant *i* at time *j*. Let *Y*_*Oi*_ and *Y*_*Mi*_ denote the vectors of observed and missing variables for participant *i*. For now, let us also assume that all the missing data are due to dropout, so that for a participant *i*, data are all observed until time point *D*_*i*_ ∈ {0, … , *J*}, and missing thereafter. So *Y*_*Oi*_ = (*Y*_*i0*_, … , *Y*_*iD*__*i*_)^T^, and *Y*_*Mi*_ = (*Y*_*iD*__*i*__+ 1_, … , *Y*_*iJ*_)^T^.

To impute the missing values, we need to define a distribution for the missing data Y_*Mi*_, given the treatment arm and observed data, that is (*Y*_*Mi*_|*Y*_*Oi*_, *D*_*i*_, *T*_*i*_). Under MAR, this distribution is independent of *D*_*i*_ and is (*Y*_*Mi*_|*Y*_*Oi*_, *T*_*i*_). Under MNAR assumptions, however, it depends on *D*_*i*_, and we need to define the distribution according to some plausible assumption. A practical option is to make statements about the unobserved data by reference to other groups of participants in the trial (typically participants in different treatment arms).

Reference‐based multiple imputation involves the following steps (Carpenter et al., 2013; Cro, Morris, Kenward, & Carpenter, 2016):

1. For each treatment arm separately, fit a multivariate normal (MVN) model for *Y*_*ij*_ using the observed data (assuming MAR).
2. Draw a mean vector and a covariance matrix from the posterior distribution of the MVN model parameters.
3. For each participant with missing data, use the draw from Step 2 to form the joint distribution of *Y*_*Oi*_ and *Y*_*Mi*_. Different assumptions can be used to construct this joint distribution (see Section 3.3).
4. For each participant, use the joint distribution to construct the conditional distribution of *Y*_*Mi*_ given *Y*_*Oi*_ and draw random values from that conditional distribution to impute the missing data.
5. Repeat Steps 2–4 *m* times to construct *m* imputed datasets.

The analysis can then be conducted as with standard multiply‐imputed datasets. That is, the parameters of interest and their variances are estimated by fitting the model of interest to each dataset and combined using Rubin's rules (Rubin, 1987). Guidance for the analysis of multiply‐imputed cost‐effectiveness data is provided elsewhere (Briggs et al., 2003; Burton et al., 2007; Faria et al., 2014; Manca & Palmer, 2005).

### 3.3 Constructing the joint distribution

**Figure 2:**
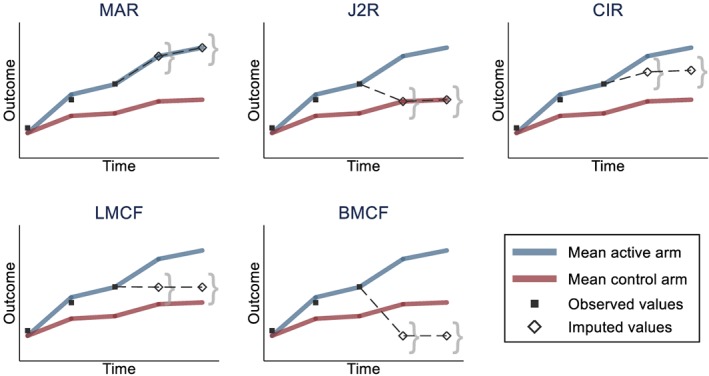
Illustration of reference‐based imputation options. Black squares are observed values for a participant in the active arm dropping out after the third time point. Hollow diamonds represent the average imputed values for that participant, and the curly brackets represent the imputation uncertainty around that average. The reference group (for J2R and CIR) is the control arm. Note that for clarity, the participant is assumed to follow closely the mean of its arm before dropout. The imputed values will actually depend on the observed data, and, for example, a participant with higher values before dropout will tend to have higher imputed values. BMCF, baseline mean carried forward; CIR, copy increments in reference; J2R, jump to reference; LMCF, last mean carried forward; MAR, missing at random [Colour figure can be viewed at http://wileyonlinelibrary.com]

Several options to construct the joint distribution of the observed and unobserved data have been proposed, each reflecting a different MNAR mechanism (Carpenter et al., 2013). The appropriate choice will be context‐specific, but here, we describe some options that may be of particular relevance to trial‐based CEA. Each of these options is illustrated in Figure 2.

*Randomised arm MAR*. The distributions of the missing and observed values, conditionally on the observed variables, are assumed to be the same. The joint distribution follows an MVN with mean and covariance corresponding to the participant's randomised arm estimates. It corresponds to the default assumption with the standard multiple imputation approach. This is the natural choice when missingness is assumed independent of the outcome, or to estimate a “de jure” (per protocol) estimand, censoring after any protocol deviation.
*Jump to reference (J2R)*. After dropout, the participant's conditional outcomes are assumed to “jump” to those of the reference group (typically the control arm). The joint distribution is an MVN model with mean parameters from the randomised arm until *D*_*i*_ and from the reference group afterward. The covariance matrix corresponds to the parameters from the randomised arm until *D*_*i*_ and to the reference group for the conditional components of the post‐dropout variables, given the pre‐dropout measurements. It corresponds to assuming that, after dropping out, participants from the active arm have the same outcomes as the reference group individuals. This is a plausible choice when the treatment effect is lost after the individual leaves the study.
*Copy increments in reference (CIR)*. After dropout, the participant's conditional outcomes mimic (parallel) the gradient from the reference group. The joint distribution mean parameters follow those from the randomised arm until dropout and the increment in mean from the reference group thereafter. The covariance matrix is formed as under J2R. This may be plausible when participants are expected to maintain the treatment benefits accrued until dropout, then follow (parallel) the outcome trajectory from the reference group after that.
*Last mean carried forward (LMCF)*. After dropout, the participant's conditional outcomes remain stable, around the mean at that last time point (from their treatment arm). The joint distribution is an MVN with mean parameters from the randomised arm until dropout, and the mean parameter from their randomised arm at time *D*_*i*_ for all the following time points. The covariance parameters follow those of the randomised arm. This is an appropriate choice when the outcomes are likely to remain stable, on average, after dropout. Note that this is distinct from the ad hoc “last observation carried forward” approach (Molenberghs et al., 2004), as values are drawn from a well‐defined posterior distribution.
*Baseline mean carried forward (BMCF)*. After dropout, the participant's conditional outcomes are assumed to “jump” back to the baseline mean level. The joint distribution is an MVN with mean parameters from the randomised arm until dropout, and the mean parameter from their randomised arm at baseline for the following time points. The covariance parameters follow those of the randomised arm. This assumption may be plausible when participants are anticipated to lose treatment benefits and return to their baseline values. Although this option was not considered in Carpenter et al. (2013), it seemed relevant in our motivating example.

Note that for J2R and CIR, we need to specify a reference group (typically the control arm). For a participant already in the reference group, the distribution will be the same as under MAR.

### 3.4 Extension to cost‐effectiveness data

In this section, we build on the original framework described above to handle key features commonly encountered in CEA.

#### 3.4.1 Handling cost and effectiveness endpoints

The MVN framework in which the algorithm is implemented can be extended naturally to accommodate additional endpoints. Although in the original model the size of the *Y*_*ij*_ vector was defined by the number of repeated measures, it can be extended to a vector of size J* = *J*_*e*_ + *J*_*c*_, where *J*_*e*_ and *J*_*c*_ capture the number of repeated measures of effectiveness and costs, respectively. This results in an MVN model defined, for each treatment arm, by a mean vector of size J*, and a variance–covariance matrix of size J* × J*. The joint distribution options described above in Section 3.3 can be logically extended for two distinct endpoints, which do not necessarily have to follow the same follow‐up measurements schedule (*J*_*e*_ ≠ *J*_*c*_) or missing data pattern. For example, with J2R, the distribution of the unobserved cost and effectiveness variables, conditional on the observed variables, can be set to follow the conditional distributions for the corresponding variables from the reference arm. Or with LMCF, it is simply the mean for the corresponding endpoint (cost or effectiveness) that is carried forward to the following time points.

#### 3.4.2 Allowing for differential missing data assumptions between endpoints

An important feature of cost‐effectiveness data is that the mechanism that gives rise to missing costs may differ from that of missing effectiveness data. For example, the data may come from different sources (e.g., case report forms versus patient‐reported questionnaires) or be collected at different time points. We may want to assume that only one of the endpoints is MNAR and that the other may be MAR. To allow for this, for each participant *i*, *Y*_*Mi*_ can be split in two vectors: *Y*_*MARi*_, consisting of the MAR‐missing variables, and *Y*_*MNARi*_, of the MNAR‐missing variables. The conditional distribution of *Y*_*MNARi*_ given *Y*_*Oi*_ and *Y*_*MARi*_ can then be defined following the options described above. The mean parameters are straightforward to derive, following the principles described in Section 3.3, with the mean parameters from the MAR‐missing variables corresponding to those from the randomised arm. For MAR, LMCF, and BMCF, the covariance matrix will be that of the randomised arm. For J2R and CIR, the covariance matrix requires some further derivation, and the technical details are reported in Data S1. Once the joint distribution has been defined, the remaining steps of the algorithm (see Section 3.2) can be followed, drawing values for (*Y*_*MARi*_, *Y*_*MNARi*_) conditionally on *Y*_*Oi*_ for each participant.

#### 3.4.3 Interim‐missing data

So far, it was assumed that missing data were monotone within each endpoint (cost or effectiveness), so that all data were missing after a given point in time. It is however common for trial‐based CEA to have interim‐missing data (an endpoint measure is missing at a particular time point but observed at a subsequent follow‐up point). We have extended the reference‐based framework to accommodate this. If the interim and dropout missing data mechanisms are the same, the joint distribution can be naturally defined. For example, with J2R, we can assume that for each individual, the missing (interim or dropout) variables follow the distribution from the reference group conditionally on the observed data. Similarly, for LMCF, the mean carried forward can be drawn from the last observation before the missing time point. However, the reasons for the interim‐missing data may differ from those of the dropout, and it will sometime seem sensible to assume that only dropout missing data are MNAR (and that the interim‐missing are MAR). In this case, the joint distribution can be built following the approach described in Section 3.4.2, with the interim‐missing data added to the vector of *Y*_*MARi*_ variables. The MNAR endpoints would then follow the specified distribution, conditionally on the observed and the interim‐missing variables.

### 3.5 Implementation in Stata

Drawing on the *mimix* Stata command (Cro et al., 2016), we developed *CEmimix*, a Stata do‐file to implement reference‐based multiple imputation for cost‐effectiveness data. The code is reported in Data S2, and available online (Leurent, Gomes, Cro, Wiles, & Carpenter, 2019). Instructions for using *CEmimix* are provided in Data S3. In brief, the user needs to specify the list of effectiveness and cost variables, the treatment arm variable, any additional imputation covariates, and the choice of imputation methods for the effectiveness and cost endpoints. The program then follows the algorithm described in Section 3.2 and returns the corresponding multiply‐imputed datasets which can be analysed using the *mi estimate* command in Stata. Optionally, it allows the user to specify different imputation methods for the interim‐missing data and to restrict the multiple imputation to a subset of participants. Further technical details are provided in the code file (Data S2) and in Cro et al. (2016).

## 4 RESULTS

In this section, we illustrate the reference‐based multiple imputation approach for assessing the sensitivity of the CoBalT cost‐effectiveness results to different missing data assumptions.

### 4.1 MAR analysis

**Figure 3:**
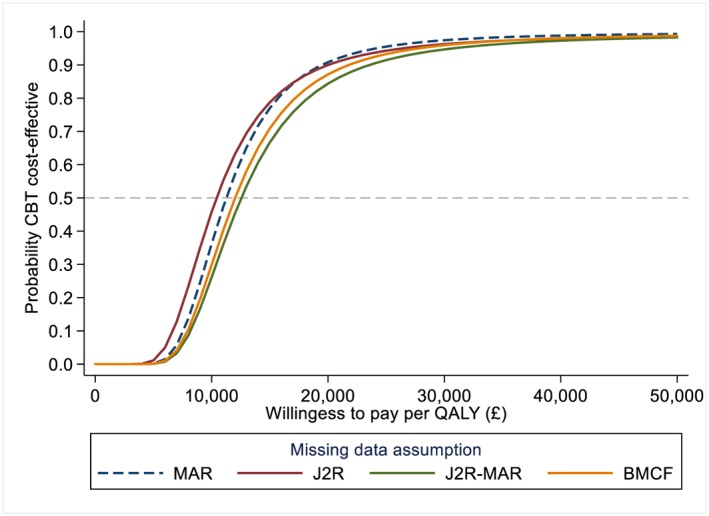
Cost‐effectiveness acceptability curve under different missing data assumptions. J2R‐interim curve not shown, similar to J2R curve. Based on N = 469 participants and *m* = 100 imputations. Based on seemingly unrelated regression of the imputed datasets, alternative approaches such as nonparametric bootstrapping could have been used (Faria et al., 2014). BMCF, baseline mean carried forward; CBT, cognitive behavioural therapy; J2R, jump to reference; MAR, missing at random; QALYs, quality‐adjusted life‐years [Colour figure can be viewed at http://wileyonlinelibrary.com]

For the base‐case analysis, we assumed that missingness was independent of the unobserved outcome values given the observed data (MAR). It is not possible to test whether this assumption holds based on the observed data, but it often constitutes a sensible starting point. Results are reported in Table 3 and Figure 3. Under MAR, participants in the CBT arm had significantly higher QALYs and costs than the usual care arm. This resulted in an ICER of £11,260 per QALY and a 90.8% probability of CBT being cost‐effective at a willingness to pay threshold of £20,000 per QALY.

**Table 3:** CoBalT reference‐based imputation results under MAR and J2R assumptions

Missing data assumption	Usual care (*N* = 235)	CBT (*N* = 234)	Difference (*N* = 469)
	*M* [95% CI]	*M* [95% CI]	*M* [95% CI]
**MAR assumption**			
QoL 6 months	0.537 [0.494, 0.581]	0.653 [0.611, 0.694]	0.115 [0.055, 0.175]
QoL 12 months	0.547 [0.498, 0.595]	0.625 [0.579, 0.671]	0.079 [0.012, 0.145]
QALYs	0.531 [0.492, 0.569]	0.619 [0.582, 0.657]	0.088 [0.035, 0.142]
Total cost (£)	803 [694, 912]	1,798 [1,641, 1,956]	996 [802, 1,190]
ICER (£/QALY)			11,260
Probability cost‐effective^a^			90.8%
**J2R assumption** b			
QoL 6 months	0.537 [0.494, 0.581]	0.640 [0.597, 0.683]	0.103 [0.042, 0.164]
QoL 12 months	0.547 [0.498, 0.595]	0.614 [0.566, 0.661]	0.067 [0.000, 0.134]
QALYs	0.531 [0.492, 0.569]	0.610 [0.572, 0.649]	0.079 [0.025, 0.134]
Total cost (£)	803 [694, 912]	1,615 [1,464, 1,767]	813 [630, 996]
ICER (£/QALY)			10,244
Probability cost‐effective^a^			90.8%

*Note*. Based on *m* = 100 imputations.

Abbreviations: CBT, cognitive behavioural therapy; ICER, incremental cost‐effectiveness ratio; J2R, jump to reference; M, mean; MAR, missing at random; QALYs, quality‐adjusted life‐years; QoL, quality of life.

a At £20,000/QALY.

b Assuming QoL and costs for dropout participants in CBT arm jump to usual care values. Interim‐missing QoL assumed to be MAR.

### 4.2 MNAR sensitivity analyses

We then conducted sensitivity analysis under different MNAR assumptions. First, it was assumed that participants dropping out from the CBT arm stopped engaging with the intervention and that their QoL and costs followed (“jumped to”) that of the control group from that point onwards (J2R). Interim‐missing data were assumed to be MAR. We can see in Table 3 how this assumption affected the mean QoL and cost estimates at the different time points. The QoL estimates in the CBT arm reduced towards the values of the usual care arm, resulting in a smaller difference in overall QALYs. Similarly, for the cost, the CBT arm costs were lower than under MAR, resulting in a smaller difference between arms. Overall, under this assumption, the ICER of CBT was slightly lower than under MAR (£10,244 per QALY), but the probability of being cost‐effective at £20,000 per QALY was unaffected (90.8%; Table 3). We can see on the cost‐effectiveness acceptability curve (Figure 3) that MAR and J2R results were relatively similar across different willingness to pay thresholds, with a probability of CBT being cost‐effective above 90% for any willingness to pay threshold above £20,000 per QALY.

We then explored the impact of further missing data assumptions, for which results are summarised in Table 4. We first conducted the same sensitivity analysis but assumed that interim‐missing QoL data also “jumped to reference” (J2R‐interim). This had little impact on the results (Table 4). We then assumed that only QoL were J2R and that costs were MAR (J2R‐MAR). This was to represent a conservative scenario (for CBT cost‐effectiveness), assuming that participants dropping out from the CBT arm “jumped to” the QoL from the usual care group but that costs would still be similar to completers in the CBT arm. Finally, we conducted a more extreme scenario where we assumed that QoL of dropout participants went back to baseline values (BMCF). Note that this is likely conservative in terms of within‐arm QALYs but not necessarily in terms of difference between arms. Although the exact estimates varied slightly under these different missing data assumptions, none significantly affected the CEA conclusions, with an ICER ranging from £10,244 to £12,552 per QALY and a probability of being cost‐effective between 84.4% and 90.8% at £20,000 per QALY (Table 4 and Figure 3). Overall, these results suggest that for any willingness to pay above £20,000 per QALY, CBT is likely to provide good value for money, and the trial CEA conclusions appear robust to various missing data mechanisms.

**Table 4:** Summary of cost‐effectiveness results under different missing data assumptions

Missing data assumption	Difference in QALYs	Difference in costs (£)	ICER (£/QALY)	Probability cost‐effective^a^
	Mean [95% CI]	Mean [95% CI]		
MAR	0.088 [0.035, 0.142]	996 [802, 1,190]	11,260	90.8%
J2R^b^	0.079 [0.025, 0.134]	813 [630, 996]	10,244	90.8%
J2R interim^c^	0.078 [0.024, 0.132]	813 [630, 996]	10,423	90.0%
J2R‐MAR^d^	0.079 [0.025, 0.134]	997 [801, 1,192]	12,552	84.4%
BMCF^e^	0.083 [0.029, 0.137]	996 [802, 1,190]	12,016	87.2%

*Note*. Based on *N* = 469 participants and *m* = 100 imputations. Note that results on the 368 participants with complete cost and effectiveness data were incremental QALYs = 0.091 (95% CI [0.032, 0.149]) and incremental costs = £1,011 (95% CI [817, 1,204]).

Abbreviations: BMCF, baseline mean carried forward; CBT, cognitive behavioural therapy; CI, confidence interval; ICER, incremental cost‐effectiveness ratio; J2R, jump to reference; M, mean; MAR, missing at random; QoL, quality of life; QALYs, quality‐adjusted life‐years.

a At £20,000/QALY.

b Assuming QoL and costs for dropout participants in CBT arm jump to usual care values. Interim‐missing QoL assumed to be MAR.

c Assuming QoL and costs for dropout participants in CBT arm jump to usual care values, but interim‐missing QoL were assumed J2R.

d Assuming QoL and costs for dropout participants in CBT arm jump to usual care values, but missing costs were assumed MAR.

e Assuming QoL for dropout participants goes back to baseline values. Missing costs and interim‐missing QoL were assumed MAR.

## 5 DISCUSSION

This study proposes a sensitivity analysis framework for addressing MNAR cost‐effectiveness data using the reference‐based multiple imputation approach. Drawing on recent work proposed to address missing clinical outcomes in longitudinal trials (Carpenter et al., 2013), our paper extends reference‐based multiple imputation to jointly handle missing cost and effectiveness endpoints and allows for features commonly seen in CEA, such as interim‐missing data. We illustrated the approach in the CoBalT trial, evaluating the cost‐effectiveness of CBT as an adjunct to pharmacotherapy for primary care patients with treatment‐resistant depression (Wiles et al., 2014). We formulated contextually plausible departures from the MAR assumption and found that the trial cost‐effectiveness conclusions were robust to varied missing data assumptions. The software code was also provided, with instructions, to facilitate the implementation of the methods.

The development of sensitivity analysis strategies for addressing potential departures from the MAR assumption in trial‐based CEA is an active area of research (Faria et al., 2014; Gabrio et al., 2018; Leurent, Gomes, Faria, et al., 2018; Mason et al., 2018). One of the key strengths of reference‐based imputation compared with other sensitivity analysis approaches is the intuitive formulation of the missing data assumptions. This matters because the main challenge when conducting missing data sensitivity analysis is to formulate assumptions that are contextually relevant and accessible to a broad audience. Although similar claims have been made using other pattern mixture models (Faria et al., 2014; Leurent, Gomes, Faria, et al., 2018; Mason et al., 2018), these typically formulate departures from MAR in terms of quantitative differences between observed and missing data, which are less straightforward to interpret. Another strength of this approach is that it can be conveniently implemented after MAR multiple imputation, which is increasingly used to address missing data in trial‐based CEA (Gabrio et al., 2017; Leurent, Gomes, & Carpenter, 2018). Although the potential of reference‐based imputation is more obvious in longitudinal trials, it is also relevant with single follow‐up trials and provides a convenient way to conduct “worst‐case”‐type scenarios while appropriately preserving the variance and imputation uncertainty.

A potential limitation of the proposed approach is that its current implementation relies on an MVN model, whereas QALYs and costs are likely to be nonnormally distributed. MVN multiple imputation is, however, recognised as robust to nonnormal data, as long as the estimators of interest are normally distributed (Bernaards, Belin, & Schafer, 2007; Lee & Carlin, 2017; Schafer, 1997; von Hippel, 2013). This is expected to be the case for most trial‐based CEA but could be an issue in small trials (say less than 50 participants per arm) or pilot studies. For an informal validation, we compared the CoBalT MVN results (under MAR) with multiple imputation by chained equations using predictive mean‐matching—which has been recommended to handle nonnormal data (White, Royston, & Wood, 2011)—and obtained very similar results (see Data S4).

The estimation of the variance parameters in reference‐based approaches has been a source of discussion (Ayele et al., 2014; Gao et al., 2017; Lu, 2014; Seaman, White, & Leacy, 2014). In particular, the model used for the imputation step differs from the one used for the analysis, an issue referred to as “incongeniality.” Although the definition of what should be the appropriate variance estimator when making assumptions about unobserved data is still an area for debate, a recent study showed that the use of Rubin's rules with reference‐based multiple imputation has a desirable “information‐anchored” property, in the sense that the amount of information lost by the missing data under MNAR is similar to the information loss caused by the missing data under MAR (Cro, Carpenter, & Kenward, 2019). It is worth noting that although multiple imputation provides a particularly convenient framework for implementation, the principles of reference‐based analysis are not necessarily tied with those of multiple imputation, and alternative frameworks have been proposed (Lu, 2014).

One key challenge of MNAR sensitivity analyses concerns the choice of plausible missing data assumptions in practice (Faria et al., 2014; Leurent, Gomes, Faria, et al., 2018). Although such assumptions are made more transparent in the proposed method, these still need to be informed by subject‐matter knowledge and discussion with relevant “experts” (e.g., trial investigators and practitioners, clinical experts, and patient representatives). The plausibility of each assumption is likely to be a matter of debate, but it is important to keep in mind that the true missing data mechanism is always unknown and that the aim of the sensitivity analyses is to indicate how results could differ under a range of plausible assumptions (Morris, Kahan, & White, 2014). Ideally, the choice and plausibility of each assumption should be prespecified, for example, in the health economic analysis plan (Dritsaki et al., 2018). If sensitivity analyses results differ importantly, investigators should draw conclusions in light of the different results and the plausibility of the respective assumptions (Leurent, Gomes, & Carpenter, 2018). An additional complexity in CEA is to formulate relevant assumptions for each endpoint in light of their differential nature. For example, the J2R assumption is generally seen as conservative for the effectiveness, as it assumes no treatment effect in those with missing data. This may not be the case for the cost endpoint as the difference is typically expected in the opposite direction (new treatment more expensive) and a J2R assumption then becomes liberal.

Although our case study clearly illustrates the principles of the reference‐based approach, it is not without limitations. For example, we worked with a limited number of variables for clarity, but additional variables, such as individual costs components could be included in the model. In addition, although the proportion of missing data was relatively modest in our study, it would be interesting to investigate the extent to which larger amount of missing data may amplify the impact of sensitivity analysis. The choice of MNAR assumption will be specific to each trial, and further examples of applications in different settings would also be of interest.

The reference‐based approach is particularly appealing due to its qualitative formulation of the MNAR assumptions, but it may not be the most appropriate approach to conduct sensitivity analysis in every trial‐based CEA. In particular, other methods have been proposed based on a “delta” parameter, capturing the plausible difference between observed and missing data (Faria et al., 2014; Leurent, Gomes, Faria, et al., 2018). This idea is relatively flexible and may be an interesting alternative when none of the reference‐based option appear contextually plausible. A fully Bayesian model has also been proposed to simultaneously handle missing data and complex data structures, particularly when external data are available (e.g., expert beliefs) to inform priors (Baio, 2014; Gabrio et al., 2018, 2019; Mason et al., 2018). Other references provide a more comprehensive review of alternative missing data methods (Molenberghs, Fitzmaurice, Kenward, Tsiatis, & Verbeke, 2014; Molenberghs & Kenward, 2007).

Reference‐based methods are still relatively novel, and there is scope for further research. Although the normality assumption has been found reasonable for multiple imputation under MAR, assessing the robustness of the proposed approach to nonnormal data in realistic settings is warranted. The approach could in theory be extended to nonnormal models, and this would be an interesting area for further development. This paper considered scenarios where one endpoint was assumed to be MAR and the other MNAR but did not allow for distinct MNAR mechanisms simultaneously (e.g., assuming the effectiveness follows J2R and the cost LMCF). Another development would be to allow for different mechanisms for different components of the endpoint. For example, assuming that self‐reported resource use items are MNAR, whereas other costs items based on medical records are MAR. Finally, this paper focused on addressing continuous outcomes, as these are most common in CEA (Leurent, Gomes, & Carpenter, 2018), but extending to other types of effectiveness measure (e.g., binary or time‐to‐event) would provide a valuable contribution.

In conclusion, this study directly addresses the lack of accessible methods for handling MNAR data in trial‐based CEA. Reference‐based multiple imputation is relatively straightforward to implement and facilitates the formulation of relevant, accessible assumptions. We hope this approach will help future CEA based on incomplete trial data to routinely conduct sensitivity analyses departing from the MAR assumption.

## CONFLICT OF INTEREST

J. C. reports grants from Medical Research Council, personal fees from Statistical consultancy for GSK, grants from Statistical consultancy for Amgen, during the conduct of the study, personal fees from Swiss Winter Epidemiology School, personal fees from Wiley, and personal fees from Springer, outside the submitted work. N. W. reports grants from University of Bristol, during the conduct of the study. B. L., M. G., and S. C. declare that they have no conflict of interest.

## Supporting information


**Data S1**. Supporting information

**Data S2**. Supporting information

**Data S3**. Supporting information

**Data S4**. Supporting information
