# Supplementary material for: Reference‐based multiple imputation for missing data sensitivity analyses in trial‐based cost‐effectiveness analysis
Source: Health Econ. 2019 Dec 17;29(2):171–84. doi: 10.1002/hec.3963 (PMC7004051; doi:10.1002/hec.3963)
Supplement: Supplementary file 1 — Data S1. Supporting information [file HEC-29-171-s001.pdf]

Appendix - Covariance matrix derivation for J2R and CIR

Let μ_A, μ_R, Σ_A, and Σ_R denote the mean vectors and covariance matrices drawn from the posterior distributions under MAR for the active and reference arm respectively. For any individual, these matrices can be partitioned according to whether the corresponding variables are observed (1), MAR-missing (2), or MNAR-missing (3).

$$\text{So that } \Sigma_A = \begin{bmatrix} A_{1,1} & A_{1,2} & A_{1,3} \\ A_{2,1} & A_{2,2} & A_{2,3} \\ A_{3,1} & A_{3,2} & A_{3,3} \end{bmatrix} \text{ and } \Sigma_R = \begin{bmatrix} R_{1,1} & R_{1,2} & R_{1,3} \\ R_{2,1} & R_{2,2} & R_{2,3} \\ R_{3,1} & R_{3,2} & R_{3,3} \end{bmatrix}$$

Let us denote

$$\begin{pmatrix} Y_1 \\ Y_2 \\ Y_3 \end{pmatrix} \sim N\left(\begin{bmatrix} \mu_1 \\ \mu_2 \\ \mu_3 \end{bmatrix}, \begin{bmatrix} \Sigma_{1,1} & \Sigma_{1,2} & \Sigma_{1,3} \\ \Sigma_{2,1} & \Sigma_{2,2} & \Sigma_{2,3} \\ \Sigma_{3,1} & \Sigma_{3,2} & \Sigma_{3,3} \end{bmatrix} \right)$$

the distribution for an individual in the active arm assumed to "jump-to-reference" for Y_3. That is we want the distribution of (Y_1, Y_2) to follow the distribution from the active arm, and the conditional distribution of Y_3 given (Y_1, Y_2) to follow the corresponding conditional distribution from the reference arm.

The distribution of (Y_1, Y_2) follows that of the active arm:

$$\begin{pmatrix} Y_1 \\ Y_2 \end{pmatrix} \sim N\left(\begin{bmatrix} \mu_{A1} \\ \mu_{A2} \end{bmatrix}, \begin{bmatrix} A_{1,1} & A_{1,2} \\ A_{2,1} & A_{2,2} \end{bmatrix} \right)$$

Let $_{12}$ denotes the joined elements of the observed and MAR-missing variables. In the reference arm the conditional distribution $[Y_3|Y_{12} = y_{12}]$ is given by

$$N\Big(\Big[\mu_{A3} + (y_{12} - \mu_{A12})^T R_{12,12}^{-1} R_{12,3}\Big], \Big[R_{3,3} - R_{3,12} R_{12,12}^{-1} R_{12,3}\Big] \Big)$$

Following the same decomposition for the jump-to-reference distribution, we have the following constraints on the covariance parameters:

$$\Sigma_{12,12} = A_{12,12} \tag{1}$$

$$\Sigma_{12,12}^{-1}\Sigma_{12,3} = R_{12,12}^{-1} R_{12,3} \tag{2}$$

$$\Sigma_{3,3} - \Sigma_{3,12}\Sigma_{12,12}^{-1}\Sigma_{12,3} = R_{3,3} - R_{3,12} R_{12,12}^{-1} R_{12,3} \tag{3}$$

Which can be solved by

$$\Sigma_{12,12} = A_{12,12} \tag{4}$$

$$\Sigma_{12,3} = A_{12,12} R_{12,12}^{-1} R_{12,3} \tag{5}$$

$$\Sigma_{3,3} = R_{3,3} - R_{3,12} R_{12,12}^{-1} (R_{12,12} - A_{12,12}) R_{12,12}^{-1} R_{12,3} \tag{6}$$

The J2R distribution is therefore:

$$\begin{pmatrix} Y_1 \\ Y_2 \\ Y_3 \end{pmatrix} \sim N\left(\begin{bmatrix} \mu_{A1} \\ \mu_{A2} \\ \mu_{R3} \end{bmatrix}, \begin{bmatrix} A_{1,1} & A_{1,2} & \Sigma_{1,3} \\ A_{2,1} & A_{2,2} & \Sigma_{2,3} \\ \Sigma_{3,1} & \Sigma_{3,2} & \Sigma_{3,3} \end{bmatrix} \right)$$

where $\Sigma_{12,3}$, $\Sigma_{3,12}$, and $\Sigma_{3,3}$ are defined in (5) and (6).

For CIR, the covariance matrix will be defined similarly, but the mean for Y_3 will be defined by the *increment* in mean from the reference arm. For MAR, LMCF, and BMCF the covariance matrix is that of the active arm.
