# Supplementary material for: Reference‐based multiple imputation for missing data sensitivity analyses in trial‐based cost‐effectiveness analysis
Source: Health Econ. 2019 Dec 17;29(2):171–84. doi: 10.1002/hec.3963 (PMC7004051; doi:10.1002/hec.3963)
Supplement: Supplementary file 2 — Data S2. Supporting information [file HEC-29-171-s002.pdf]

```

*****************************************************************
*** REFERENCE-BASED IMPUTATION OF COST-EFFECTIVENESS DATA ***
*****************************************************************

***********************************************************************
//Stata program to conduct reference-based multiple imputation with cost-effectiveness data
//See accompanying instructions to use this do-file

* Version: 1.0
* Date: 19 December 2018
* Author: Baptiste Leurent, LSHTM. Based on mimix.ado by Cro et al. (Stata J. 2016 16(2):443-463)
* Stata version: 15

** CONTENT:
    * I  -  SET-UP
    * II -  DEFINE ROUTINES
    * III - PREPARE DATA FOR IMPUTATION
    * IV -  RUN MVN
    * V  -  MNAR IMPUTATION, FOR EACH ARM AND PATTERN
    * VI -  SAVE AS MI DATASET

***********************************************************************


***********************
***  I - SET-UP     ***
***********************
//Define here program parameters (dataset,variable names, imputation method, etc.)
//See do-file instructions

    macro drop _all

** Parameters **

    * Required
        global m   //Number of imputations
        global emethod    //MAR J2R CIR LMCF BMCF
        global cmethod    //MAR J2R CIR LMCF BMCF

        global data
        global effectv
        global costv
        global covariates
        global idv
        global treatv
        global refgroup

    *Options
        global interimMAR   // effect, cost, or leave blank
        global restrictto  //Restrict MNAR imputation to a specific subgroup (e.g. arm==1). Leave blank otherwise.
        global seed  //Specify seed for reproducibility. Leave blank for random seed
        global saving


** Check parameters
    //Basic error checks

    if !inlist("$emethod","MAR","J2R","CIR","LMCF","BMCF") | !inlist("$cmethod","MAR","J2R","CIR","LMCF","BMCF") {
        display as error "Please specify imputation method for effect and cost: MAR J2R CIR LMCF or BMCF"
        exit
        }
    if inlist("$emethod","J2R","CIR","LMCF","BMCF") & inlist("$cmethod","J2R","CIR","LMCF","BMCF") & "$emethod"!="$cmethod" {
        display as error "Different MNAR mechanisms for effect and costs not allowed"
        exit
        }
    if !strpos("$interimMAR", "effect") & !strpos("$interimMAR", "cost") & "$interimMAR"!="" {
        display as error " 'interimMAR' should be 'effect', 'cost', or nothing"
        exit
        }
    if (inlist("$emethod","J2R","CIR") | inlist("$cmethod","J2R","CIR")) & "$refgroup"=="" {
        display as error "Please specify reference group for CIR or J2R"
        exit
        }
    if "$idv"=="" | "$treatv"=="" {
        display as error "Please specify treatment arm and patient identifier variables"
        exit
        }



*********************************
***  II - DEFINE ROUTINES     ***
*********************************
    //Define Mata functions used in imputation step

        mata: mata clear

** Mata functions to manipulate list of variables
    mata
```

```
        // Common : Returns common elements between 2 vectors
            real vector common(real vector V1, real vector V2)
            {
                st_local("v1",invtokens(strofreal(V1)))
                st_local("v2",invtokens(strofreal(V2)))
                stata("local l2: list v1 & v2")
                res=strtoreal(tokens(st_local("l2")))
                return(res)
            }
        // Join: Returns elements in either of 2 vectors
            real vector join(real vector V1, real vector V2)
            {
                st_local("v1",invtokens(strofreal(V1)))
                st_local("v2",invtokens(strofreal(V2)))
                stata("local l2: list v1 | v2")
                res=sort(strtoreal(tokens(st_local("l2")))',1)'
                return(res)
            }
        // Exclude: Returns elements of V1, not contained in V2.
            real vector exclude(real vector V1, real vector V2)
            {
                st_local("v1",invtokens(strofreal(V1)))
                st_local("v2",invtokens(strofreal(V2)))
                stata("local l2: list v1 - v2")
                res=sort(strtoreal(tokens(st_local("l2")))',1)'
                return(res)
            }
    end

** Mata function to build conditional covariance matrix
    //Used for J2R and CIR imputation
    //Build joint covariance matrix, so that MNAR-missing variables follow distribution from reference arm, conditionally on observed or MAR-missing variables
    //Parameters = covariance matrix in active arm; covariance in reference arm; indicator of observed or MAR variables; indicator of MNAR-missing varibales
    //See technical details in Appendix
    mata
        real matrix condcov(real matrix SigmaA, real matrix SigmaR, real vector vobsmar, real vector vmnar)
            {
            A11 = SigmaA[vobsmar,vobsmar]   //Decompose var/covar in active and reference arm
            R11 = SigmaR[vobsmar,vobsmar]
            R12 = SigmaR[vobsmar,vmnar]
            R22 = SigmaR[vmnar,vmnar]
            J11=A11  //Solve contraints (see Appendix)
            J12=A11*invsym(R11)*R12
            J22=R22-(R12)'*invsym(R11)*(R11-A11)*invsym(R11)*R12
            J = J(cols(SigmaA),cols(SigmaA),.)  //Build joint covariance matrix
            J[vobsmar,vobsmar]=J11
            J[vobsmar,vmnar]=J12
            J[vmnar,vobsmar]=J12'
            J[vmnar,vmnar]=J22
        return(J)
        }
    end



*******************************************
*** III - PREPARE DATA FOR IMPUTATION    ***
*******************************************

*** Open original dataset
    use "$data" , clear
    describe
    list in 1/5, noobs

*** Prepare macros and variables for program

    **Global macros
        *Seed
            capture: set seed $seed
                //Affect random draws in MVN, and imputation steps. Will obtain same draws with same sorted data (and do file)
        *Outcomes list
            global responses $effectv $costv
        *Number of variables
            global nresp: word count $responses
            global ncov: word count $covariates
            global nvar = $nresp + $ncov  //"nct" in mimix
        *Number of treatment group
            tab $treatv
            global ntreat = r(r)
        *First effectiveness and cost variable
            //Note: in mata, variables order always $effectv $costv $covariates
            global v1effect=1
            local neff: word count $effectv
            global v1cost= `neff' + 1  // Cost var = first after effectiveness vars.
        *Interim-MAR option
            if strpos("$interimMAR", "effect") global eintmeth iMAR
                else global eintmeth $emethod
            if strpos("$interimMAR", "cost") global cintmeth iMAR
                else global cintmeth $cmethod
```

```
        *Check
            macro list

    ** New variables
        *Treatment arm variable
            egen m_treat=group($treatv)  //Recoding = 1,2,..
            tab m_treat
        *New reference-group code
            if "$refgroup"!="" sum m_treat if $treatv==$refgroup
            global m_refer=r(max)
            display "New reference arm code = $m_refer"
        *Observation ID
            if substr("`:type $idv'",1,3)=="str" encode($idv), generate(m_id)
            else gen m_id = $idv
            duplicates report m_id
        *Missing data pattern
            qui: generate m_pattern = 0
            local i=0
            foreach var of varlist $responses  {
                local k2 = 2^(`i++')    //Will assign a unique number by pattern, for any number of variables.
                qui: replace m_pattern = m_pattern + `k2'  if `var'== .
                }
            tab m_pattern m_treat ,m
        *MNAR subgroup
            //If restrictto specified, restrict MNAR imputation to these observations
            gen m_allmar=0
            if "$restrictto"!="" replace m_allmar= !($restrictto) // AllMAR=1 if restricto specified and observation not in "restrictto" subgroup
            qui: count if m_allmar==0
            if "$restrictto"!="" display "MNAR imputation restricted to `r(N)' observations out of " _N
            if `r(N)'==0  display as error "No observations MNAR-imputed - Check 'restrictto' option"

*** Sort and save
    *Save dataset
        //Original dataset + programming variables. Will be used to merge with imputed data at the end
        sort m_id
        compress
        save "originalext.dta", replace

    *Save reduced version for imputation
        keep m_id m_treat $responses $covariates m_pattern m_allmar
        order m_id m_treat $responses $covariates m_pattern m_allmar
        sort m_treat m_pattern m_allmar m_id //Sort by treat arm, missing data pattern, then PID.
        compress
        save "m_d2.dta", replace


*************************
***  IV -  RUN MVN    ***
*************************
 // Fit a multivariate normal model to the observed data, for each arm
 // Then draw mean/covariance parameters from their posterior distribution


** Set-up MCMC burn-in parameters
    local burnin = 100  //Number of iterations for the initial burn-in period
    local burnbetween 100  //Number of iterations between imputation
    local burninM = `burnin' + (($m-1)*`burnbetween')  //Total number of iterations

*** Run MVN for each treament arm, and save parameters
     forvalues i = 1/$ntreat {
        **Set-up
            use "m_d2.dta" if m_treat == `i', clear
            mi set wide  //Wide faster, can set to mlong if size error
            qui: mi register imputed $responses $covariates

        **MVN
            display as text "Performing imputation procedure for arm " as result "`i'" as text " of " as result "$ntreat" as text "..."
            mi impute mvn $responses $covariates , mcmconly burnin(`burninM')  prior(jeffreys) initmcmc(em, iter(1000)) saveptrace(mimix_parms_a`i', replace)
                //Note: Used only to fit MVN model and save trace, not doing imputation.

        **Save parameters
            //Using values from the MCMC trace. Saving every 'burnbetween' iteration is like doing random draws from from posterior distribution of the parameters

            *Open trace
                mi ptrace describe mimix_parms_a`i'
                mi ptrace use mimix_parms_a`i', clear

            *Save every 100 iterations:
                local burn = `burnin' - 1
                drop in 1/`burn'
                keep if !mod(_n-1,`burnbetween')
                generate m_treat = `i'
                drop m iter
                capture mata: mata drop mimix_all
                mata: mimix_all= st_data( ., .) //Copy dataset (all params, m_treat) into mimix_all
            *Save mean and covariance in matrices, for each m:
                forvalues k=1/$m {
                    display _n " Draw for group `i', imputation `k' "
```

```
                            *Save mean matrice:
                                mata: mean_group`i'_imp`k' = mimix_all[`k',1..$nvar]
                                    *mata: mean_group`i'_imp`k'
                            *Save covariance matrices:
                                mata: mata_VAR_group`i'_imp`k'=J($nvar,$nvar,0)
                                local step = $nvar+ 1
                                forvalues r = 1/$nvar {
                                    forvalues j = 1/$nvar{
                                        if `j' <= `r' {
                                            mata: mata_VAR_group`i'_imp`k'[`r', `j'] = mimix_all[`k', `step']
                                            local step = `step' + 1
                                        }
                                    }
                                }
                                mata: mata_VAR_group`i'_imp`k' = makesymmetric(mata_VAR_group`i'_imp`k')
                                    *mata: mata_VAR_group`i'_imp`k'
                        } //End of saving mean and cov matrices

            }  //End of MVN loop.



    ******************************************************************
    ***  MNAR IMPUTATION, FOR EACH ARM AND MISSIGN DATA PATTERN   ***
    ******************************************************************

    **** Set up

        ** Describe data
            use "m_d2.dta", clear
            describe
            tab m_pattern m_treat,m

        ** Save characteristics of each arm+pattern group

            *First and last observation
                gen n=_n
                bysort m_treat m_pattern m_allmar: gen nfirst=n[1]
                bysort m_treat m_pattern m_allmar: gen nlast=n[_N]
            *Number of missing var
                egen nmiss=rowmiss($responses $covariates)
            *Contract
                contract m_treat m_pattern m_allmar nfirst nlast nmiss
                rename _freq ncount
                gen groupID=_n
            *Order var and save in a matrix
                mkmat m_treat m_pattern m_allmar ncount nfirst nlast nmiss groupID , mat(m_group)
                matrix list m_group
            *Save number of combinations/groups
                global max_indicator=_N

        ** Indicator of effect/cost/MAR/MNAR variables
                mata: mata_responses=J(1,0,.)
                mata: mata_eff=J(1,0,.)
                mata: mata_cost=J(1,0,.)
                mata: mata_meth_mar=J(1,0,.)
                mata: mata_meth_mnar=J(1,0,.)
                local j=0
                foreach var in $responses $covariates {  //Note: Variables identified by their position, use always same order
                    local j=`j'+1
                    if strpos("$responses","`var'")  mata: mata_responses=(mata_responses,`j')
                    if strpos("$effectv","`var'")  mata: mata_eff=(mata_eff,`j')
                    if strpos("$costv","`var'")    mata: mata_cost=(mata_cost,`j')
                    if strpos("$effectv","`var'")*("$emethod"=="MAR") | strpos("$costv","`var'")*("$cmethod"=="MAR") {
                        mata: mata_meth_mar=(mata_meth_mar,`j')
                        }
                    if strpos("$effectv","`var'")*("$emethod"!="MAR") | strpos("$costv","`var'")*("$cmethod"!="MAR") {
                        mata: mata_meth_mnar=(mata_meth_mnar,`j')
                        }
                    }


        ** Empty matrix to save imputed data
            global new_varlist m_treat m $responses $covariates m_id  //List of variables to be saved after each mata-imputation (used when converting back to Stata)
            mata: mata_all_new=J(0,$nvar+3,.) // Size= nvar+3(treat,m,ID)


    **** Begining of "for each imputation group" loop
        //Split data in imputation groups ( = arm + missing data pattern).
        //For each group do:  1) Build joint distribution from MAR parameters 2) Draw missing values from that distribution 3) Redo 1-2 m times.
        //Note: large loop, encompasses "foreach imputation" loop, see below.

        forvalues i= 1/$max_indicator {  //For each imputation group

            display _n "--- Imputation for group `i' of $max_indicator ---"

            ** Set up
                //Group charateristics, before going into "for each m" loop.

```

```
        *Save group characteristics
            matrix list m_group
            local trt_grp= m_group[`i',1]
            local pattern = m_group[`i',2]
            local allmar= m_group[`i',3]
            local ncount= m_group[`i',4]
            local nfirst= m_group[`i',5]
            local nlast= m_group[`i',6]
            local miss_count= m_group[`i',7]
            local refer = $m_refer  //Note: reference arm currently same for everyone, but allow to change if needed.

        *Indicator of complete/missing var
            qui: use m_d2.dta, clear
            mata: mata_miss = J(1,0,.)
            mata: mata_nonmiss = J(1,0,.)
            local j=0
            foreach var of varlist $responses  $covariates {
                local j=`j'+1
                if (`var'[`nfirst']>=.)  mata: mata_miss=(mata_miss,`j')
                    else  mata: mata_nonmiss=(mata_nonmiss,`j')
                }

        *Indicator of interim-MAR missing
            *Last observed cost/effect:
                mata: st_numscalar("lastobse",rowmax((common(mata_eff,mata_nonmiss),0))) //Adding a 0 so is "0" if empty matrix
                mata: st_numscalar("lastobsc",rowmax((common(mata_cost,mata_nonmiss),0))) //Adding a 0 so is "0" if empty matrix
            *Testing whether interim (+MAR option specified), for each missing variable:
                mata: st_local("misslist",invtokens(strofreal(mata_miss)))
                mata: mata_int_mar = J(1,0,.)
                foreach v of local misslist {
                    if (`v'>=$v1effect & `v'<lastobse & "$eintmeth"=="iMAR" )  | (`v'>=$v1cost & `v'<lastobsc & "$cintmeth"=="iMAR"){
                        mata: mata_int_mar=(mata_int_mar,`v')
                        }
                    }
            *Check
                mata: mata_int_mar

        *Indicator of forced-MAR variables
            //If "restricto" specified, impute all var under MAR for observations not in that subgroup.
            if `allmar'==1 mata: mata_allmar=mata_responses
            else mata: mata_allmar=J(1,0,.)

        *Identify MAR-missing variables
            //Variable is MAR if either i)Main imputation-method for that endpoint = MAR or ii) is interim-MAR or iii) observation not in "restrictto" subgroup
            //Note: use mata "common" and "join" functions defined above
            mata: mata_mar2=join(mata_meth_mar,join(mata_int_mar,mata_allmar))
            mata: mata_marmiss=common(mata_mar2,mata_miss) // MAR and actually missing. Will be those MAR-imputed for that pattern.

        *Identify MNAR-missing variables
            //Is MNAR if main imputation method=MNAR, except if i) interim-MAR missing or ii) observation not in "restrictto" subgroup
            mata: mata_mnar2=exclude(mata_meth_mnar,join(mata_int_mar,mata_allmar))
            mata: mata_mnarmiss=common(mata_mnar2,mata_miss) // MNAR and actually missing. Will be those MNAR-imputed for that pattern.

        *Indicator of any MNAR missing variables:
            mata: st_local("n_mnar_miss",strofreal(cols(mata_mnarmiss)))

        *Check all indicators:
            display as txt _n "Variables imputation status for group `i' (var numbered in order of: effect,cost,covariates)"
            display as txt "Observed:"
                mata: mata_nonmiss
            display as txt "MAR-missing:"
                mata: mata_marmiss
            display as txt "MNAR-missing:"
                mata: mata_mnarmiss

        *Save observed data
            //Save responses,covariates,ID in a mata matrix
            qui: use m_d2.dta, clear
            qui: keep in `nfirst'/`nlast'
            keep $responses $covariates m_id
            order $responses $covariates m_id
            mata: mata_obs= st_data( . , .)


    *** Begining "for each imputation" loop

        forvalues imp = 1/$m {
                display "." _cont

            ** If no missing data, copy data directly
                if `miss_count' == 0 {
                    if `imp'==1 dis "No missing"
                    *Copy observed data
```

```
                    mata: mata_new = (J(`ncount',1,`trt_grp'), J(`ncount',1, `imp'), mata_obs)  //Dataset with Arm + imp_number + observed data
                *Append to existing
                    mata: mata_all_new = (mata_all_new \ mata_new)
                }

        ** If missing data, build the joint distribution (mean vector, and covariance matrix)
            else {
                *All MAR
                    if `n_mnar_miss'==0  {  // No MNAR missing
                        if `imp'==1 dis "Imputation (Method = MAR)"
                        mata: mata_Meansv=mean_group`trt_grp'_imp`imp'
                        mata: Sigma = mata_VAR_group`trt_grp'_imp`imp'
                        }
                *J2R
                    if (`n_mnar_miss'>0) & ("$emethod" == "J2R" | "$cmethod" == "J2R")  {  // Cost or effectiveness is J2R
                        if `imp'==1 dis "Imputation (Method = J2R)"
                        *Mean
                            mata: mata_Meansv=mean_group`trt_grp'_imp`imp'
                            mata: mata_Meansv[1,mata_mnarmiss]=mean_group`refer'_imp`imp'[1,mata_mnarmiss]  //Replacing Mean from reference group for MNAR variables
                        *Covariance
                            mata: mata_nonmiss_marmiss=join(mata_nonmiss,mata_marmiss)  //Observed or MAR-missing variables.
                            mata: Sigma=condcov(mata_VAR_group`trt_grp'_imp`imp', mata_VAR_group`refer'_imp`imp',mata_nonmiss_marmiss,mata_mnarmiss)
                        }
                *CIR
                    if (`n_mnar_miss'>0) & ("$emethod" == "CIR" | "$cmethod" == "CIR")  {  //Cost or effectiveness is CIR
                        if `imp'==1 dis "Imputation (Method = CIR)"
                            **Mean
                                mata: mata_Meansv=mean_group`trt_grp'_imp`imp'
                                mata: MeansC=mean_group`refer'_imp`imp'
                                *Effect
                                    mata: mata_mnarmiss_e=common(mata_mnarmiss,mata_eff) // Effectiveness var MNAR-missing
                                    mata: st_local("vlist",invtokens(strofreal(mata_mnarmiss_e)))
                                    foreach v of local vlist {
                                        if `v'==$v1effect mata: mata_Meansv[1,`v'] = MeansC[1,`v']  //If first var missing, copy from reference arm
                                        else mata: mata_Meansv[1,`v'] = mata_Meansv[1,`v'-1] + (MeansC[1,`v']-MeansC[1,`v'-1])  //Previous mean (in current arm) + increment in mean in refer group
                                        }
                                *Cost
                                    mata: mata_mnarmiss_c=common(mata_mnarmiss,mata_cost)
                                    mata: st_local("vlist",invtokens(strofreal(mata_mnarmiss_c)))
                                    foreach v of local vlist {
                                        if `v'==$v1cost mata: mata_Meansv[1,`v'] =  MeansC[1,`v']
                                        else mata: mata_Meansv[1,`v'] = mata_Meansv[1,`v'-1] + (MeansC[1,`v']-MeansC[1,`v'-1])
                                        }
                            **Covariance
                                mata: mata_nonmiss_marmiss=join(mata_nonmiss,mata_marmiss)  //Observed or MAR-missing variables.
                                mata: Sigma=condcov(mata_VAR_group`trt_grp'_imp`imp', mata_VAR_group`refer'_imp`imp',mata_nonmiss_marmiss,mata_mnarmiss)
                        }
                *LMCF
                    if (`n_mnar_miss'>0) & ("$emethod" == "LMCF" | "$cmethod" == "LMCF") { //Cost or effectiveness is LMCF
                        if `imp'==1 dis "Imputation (Method = LMCF)"
                        *Mean
                            mata: mata_Meansv=mean_group`trt_grp'_imp`imp'
                            *Effect
                                mata: mata_mnarmiss_e=common(mata_mnarmiss,mata_eff) // Effectiveness variables MNAR-missing
                                mata: st_local("vlist",invtokens(strofreal(mata_mnarmiss_e)))
                                foreach v of local vlist {
                                    if `v'>$v1effect {  //Note: if first var missing, use the mean
                                        mata: mata_Meansv[1,`v'] = mata_Meansv[1,`v'-1]     // Copying previous mean
                                    }
                                }
                            *Cost
                                mata: mata_mnarmiss_c=common(mata_mnarmiss,mata_cost)
                                mata: st_local("vlist",invtokens(strofreal(mata_mnarmiss_c)))
                                foreach v of local vlist {
                                    if `v'>$v1cost {
                                        mata: mata_Meansv[1,`v'] = mata_Meansv[1,`v'-1]
                                    }
                                }
                        *Covariance
                            mata: Sigma = mata_VAR_group`trt_grp'_imp`imp'  //Using MAR covariance from that arm
                        }
                *BMCF
                    if (`n_mnar_miss'>0) & ("$emethod" == "BMCF" | "$cmethod" == "BMCF") { //Cost or effectiveness is BMCF
                        if `imp'==1 dis "Imputation (Method = BMCF)"
                        *Mean
```

```
                                    mata: mata_Meansv=mean_group`trt_grp'_imp`imp'
                                    *Effect
                                        mata: mata_mnarmiss_e=common(mata_mnarmiss,mata_eff) // Effectiveness variables MNAR-missing
                                        mata: st_local("vlist",invtokens(strofreal(mata_mnarmiss_e)))
                                        foreach v of local vlist {
                                            mata: mata_Meansv[1,`v'] = mata_Meansv[1,$v1effect]  // Copying mean of first variable
                                        }
                                    *Cost
                                        mata: mata_mnarmiss_c=common(mata_mnarmiss,mata_cost)
                                        mata: st_local("vlist",invtokens(strofreal(mata_mnarmiss_c)))
                                        foreach v of local vlist {
                                                mata: mata_Meansv[1,`v'] = mata_Meansv[1,$v1cost ]
                                        }
                                *Covariance
                                    mata: Sigma = mata_VAR_group`trt_grp'_imp`imp'  //Using MAR covariance from that arm
                                }

                    **Check joint distribution
                        *mata: mata_Meansv
                        *mata: Sigma

                    ** Perform imputation
                        * Expand mean vector to n observations
                            mata: mata_Means=J(`ncount', 1, mata_Meansv)
                        * Decompose the covariance matrix observed/missing
                            mata: S11 = Sigma[mata_nonmiss, mata_nonmiss]   //Covariance observed var.
                            mata: S12 = Sigma[mata_nonmiss, mata_miss] //Covariance for observed(row)Xmissing(col) var
                            mata: S22 = Sigma[mata_miss, mata_miss]  //Covariances missing var
                        *Draw missing values conditionally on observed
                            mata: m1=mata_Means[., mata_nonmiss]  //Mean param for all observed var (n times)
                            mata: m2=mata_Means[., mata_miss]  //Mean param for all missing var (n times)
                            mata: raw1=mata_obs[., mata_nonmiss] //Observed values matrix.
                            mata: meanval = m2 + (raw1 - m1)*invsym(S11)*S12 //Expectation given observed values.
                            mata:conds=S22-S12'*invsym(S11)*S12
                            mata: U = cholesky(conds)
                            mata: Z = invnormal(uniform(`ncount',`miss_count'))  //Drawn n*nmiss standard normal
                            mata: mata_y1 = meanval + Z*U'  //Draw n X nmiss following N((cond mean),Covar). = Imputed values.
                        *Merge all variables
                            mata: mata_new =J(`ncount',$nvar,.)  //Empty mat n*nvar
                            mata: mata_new[.,mata_nonmiss] = mata_obs[.,mata_nonmiss] //Add observed val
                            mata: mata_new[.,mata_miss] = mata_y1[.,.] //Add imputed val
                            mata: GI=J(`ncount',1,`trt_grp')  //Treatment group
                            mata: II=J(`ncount',1,`imp')   //Imputation number
                            mata: ID = mata_obs[.,cols(mata_obs)] //Last column of mata_obs = ID
                            mata: mata_new=(GI, II, mata_new, ID)
                        *Append to existing data
                            mata: mata_all_new = (mata_all_new \ mata_new)

                    } //End of "if missing" loop.

                } //End of "for m" loop

    } //End of "for each group" loop


*** Check data
    clear
    getmata($new_varlist)=mata_all_new
    describe
    list in 1/5, header noobs
    count
    dis _N/$m //Check same number of obs as original dataset


*********************************
***** SAVE AS MI DATASET     ****
*********************************

    *Prepare imputed data
        clear
        getmata($new_varlist)=mata_all_new  //Convert mata "all_new" to Stata
        keep $responses m m_id  //Other var will be in original dataset
        sort m_id m
        tempfile imputedv
        save `imputedv', replace

    *Add other variables from original dataset
        use originalext.dta, clear
        count
        sort m_id
        merge 1:m m_id using `imputedv', nogen update
        count //OK, N*$m

    *Add _m=0 (=observed data)
        append using originalext.dta
        replace m=0 if m==.

```

```
    *Convert to MI format
        mi import flong , m(m) id($idv) clear
        mi register imputed $responses $covariates
        mi describe
        list in 1/5

    *Clean and save
        describe
        drop m_treat-m  //Drop programming var
        sort $idv _mi_m
        list in 1/10, sepby($idv)
        compress
        label data "Reference-based imputed ($emethod-$cmethod) - `c(current_date)'"
        save "$saving", replace     //! Will overwrite dataset if already exist.

    ** Delete temporary datasets
        //Temporary datasets created for the program
        erase originalext.dta
        erase m_d2.dta
        forvalues i = 1/$ntreat {
            erase mimix_parms_a`i'.stptrace
            }

***** END *****
```
