# Supplementary material for: Reference‐based multiple imputation for missing data sensitivity analyses in trial‐based cost‐effectiveness analysis
Source: Health Econ. 2019 Dec 17;29(2):171–84. doi: 10.1002/hec.3963 (PMC7004051; doi:10.1002/hec.3963)
Supplement: Supplementary file 3 — Data S3. Supporting information [file HEC-29-171-s003.pdf]

Supporting Information 3

Using CEmimix.do

I) <u>Stata version</u>

The do-file needs Stata software[1] to run. It was developed with Stata version 15, and should work with subsequent versions (type "`version 15`" at beginning of execution). It may work with other recent versions but not earlier than 11, when `mi impute` was implemented.

II) <u>Data input format</u>

The input dataset needs to be store in Stata 'wide' format. That is, with a single record per participant. The effectiveness and cost data should be stored as separate variables for each time-point. It also needs to include a treatment arm and patient identifier variable.

III) <u>Set up</u>

The user will need to define the following global macros at the beginning of the do-file:

<u>Required information:</u>

m: The number of imputation

emethod: Imputation method for the effectiveness variable. Should be one of **MAR**, **J2R**, **CIR**, **LMCF**, or **BMCF**

cmethod: Imputation method for the cost variable. Should be one of **MAR**, **J2R**, **CIR**, **LMCF**, or **BMCF**

data: Name of dataset to be imputed. Can use path if dataset is not in working directory. E.g. `"Data\mydata.dta"`

effectv: Effectiveness variable(s), in chronological order[2]. E.g. `eq5d0 eq5d6 eq5d12`

costv: Cost variable(s), in chronological order. E.g. `tcost0 tcost6 tcost12`

idv : Unique patient identifier variable. Can be either numerical or string.

treatv : Trial treatment arm variable. Need to be numerical. Multiple arms are allowed.

<u>Optional:</u>

[1] StataCorp. 2017. Stata Statistical Software: Release 15. College Station, TX: StataCorp LLC
[2] The temporal order of the variables is used for CIR, LMCF, BMCF, and for defining interim-missing. For BMCF, the first variable need to be the baseline measurement.

refgroup : Reference group number (as coded in *treatv*). Need to be specified for J2R and CIR, can be left blank otherwise.

interimMAR: Whether interim-missing data should be imputed under MAR. Can be **cost** and/or **effect**. If not specified, uses the imputation method specified above.

covariates: Additional variables used in the imputation model. Covariates need to be complete[3] and numerical. Categorical variables need to be split in dummy (binary) variables.

seed: Specifies the seed for the random number generator. If a seed is specified (and data are sorted in same order), the program will return the same results on separate runs. If nothing is specified, a random seed is used and different runs will result in a different set of imputed data.

restrictto: Specify a subgroup on which to restrict the reference-based imputation. Other individuals are imputed under MAR. For example, could restrict MNAR assumption to a single arm or/and to participants who dropped-out for a specific reason. E.g. `(arm==1 & reason=="withdraw")`.

saving: Specify file name to save imputed dataset. Note that it will overwrite file with same name if already exist.

This do-file was primarily designed to be run on its' own once these macros have been defined. If needed, the remaining of the do-file can be modified to accommodate particular situations (e.g. other outcome types, or changing the number of burn-in iterations in the imputations) but this is not covered here.

IV) Output

On completion, CEmimix returns a multiply-imputed dataset corresponding to the options specified. If 'saving' was specified, the dataset is also saved in corresponding folder. This dataset is in Stata `mi flong` format, and can be analysed with the `mi:` family of commands. Note that CEmimix only returns a dataset and does not conduct any analysis. An example of analysis can be seen below.

V) Example

Here is an example of CEmimix syntax for CoBalT study, under J2R assumptions for effectiveness and costs. Interim missing are assumed MAR, and 100 imputations are performed.

```
global m  100            //Number of imputations
global emethod  J2R      //MAR J2R CIR LMCF BMCF
global cmethod  J2R      //MAR J2R CIR LMCF BMCF

global data  COBALT.dta
global effectv  eq5d0 eq5d6 eq5d12
global costv  tcost
global covariates  age sex
global idv  ptidno
global treatv  arm
```

[3] If a baseline covariate is not fully observed, it could be mean-imputed beforehand (White & Thompson, *Statistics in medicine*, *24*(7), pp.993-1007)

```
global refgroup  0

global interimMAR  effect cost  // effect, cost, or leave blank
global restrictto    //Restrict MNAR imputation to a specific subgroup
global seed //Specify seed for reproducibility. Leave blank for random seed
global saving  COBALT_imputed_J2R.dta
```

And an example of analysis of the resulting dataset:

```
use COBALT_imputed_J2R.dta, clear
*Calculate imputed QALYs
       mi passive: gen qaly=0.5*(eq5d0+eq5d6)/2 +0.5*(eq5d6+eq5d12)/2
*Incremental QALYs and costs
       mi estimate: regress qaly arm
       local incq=el(r(table),1,1)
       mi estimate: regress tcost arm
       local incc=el(r(table),1,1)
*ICER
       display "ICER = " `incc'/`incq'
*Probability cost effective
       gen inb20=qaly*20000-tcost
       mi estimate: regress inb20 arm
       local pce = normal(el(r(table),1,1)/el(r(table),2,1) )
       display "Probability cost effective = " round(`pce'*100,0.1) "%"
```
