# Supplementary material for: Reference‐based multiple imputation for missing data sensitivity analyses in trial‐based cost‐effectiveness analysis
Source: Health Econ. 2019 Dec 17;29(2):171–84. doi: 10.1002/hec.3963 (PMC7004051; doi:10.1002/hec.3963)
Supplement: Supplementary file 4 — Data S4. Supporting information [file HEC-29-171-s004.pdf]

Supporting Information 4

Variables distribution and comparison of imputation methods

I) <u>CoBalT variables distribution</u>

Histogram of CoBalT quality-of-life and cost variables. Based on observed data. See main manuscript for additional information.

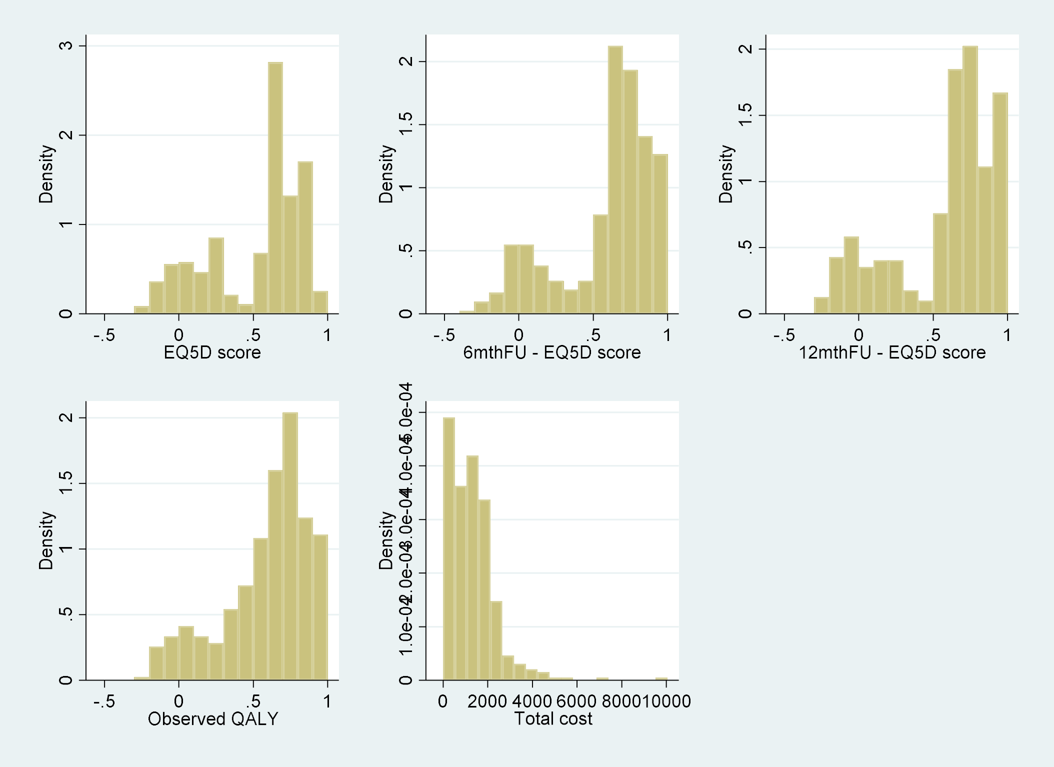

II) <u>Comparison of multivariate-normal and chained equations imputation</u>

Illustrative results to compare multivariate-normal (MVN) and chained-equations (ICE) multiple-imputation results.

Methods outline:

- Imputation conducted under missing-at-random (MAR) assumption
- Multiple imputation model as described in main manuscript. 100 imputations were performed, separately by arm. Conducted in Stata, version 15.
- MVN multiple imputation conducted using 'mi impute mvn' command (corresponding to the CEmimix model, under MAR).
  - Syntax: `mi impute mvn eq5d6 eq5d12 tcost = eq5d0 age sex bditot, add(100) prior(jeffreys) by(arm)`

- Chained-equations multiple imputation implemented with the ‘mi impute chained’ command, using predictive mean-matching[1] with 10 donors.
    - Syntax: `mi impute chained (pmm, knn(10)) eq5d6 eq5d12 tcost = eq5d0 age sex bditot,  add(100) by(arm)`
- Imputation was first conducted on the complete dataset, then restricted to the 50 first participant per arm, to assess robustness of MVN vs. ICE with a smaller sample

TABLE S3 Cost-effectiveness results with different multiple-imputation methods

Method	Difference in QALYs Mean [95%CI]	Difference in costs (£) Mean [95%CI]	ICER (£/QALY)	Probability cost-effective†
**All sample (N=469)**				
MVN	0.088 [0.034 to 0.141]	1005 [807 to 1203]	11,454	90.2%
ICE-PMM	0.088 [0.035 to 0.142]	1018 [832 to 1205]	11,543	90.1%
**N=100***				
MVN	0.125 [-0.008 to 0.258]	1242 [665 to 1820]	9,942	80.8%
ICE-PMM	0.125 [-0.005 to 0.254]	1321 [819 to 1823]	10,571	80.2%

Note. MVN=Multivariate-Normal imputation; ICE-PMM= Imputation by chained-equations with predictive-mean matching

†at £20,000/QALY.

* Analysis restricted to the first 50 participants per arm, to compare MVN to ICE-PMM results with a smaller sample size. These results are not for any inference, but only for illustration.

Notes:

1) These result are not for any inference, but only for illustration.
2) These results do not allow to draw any conclusion on which method is ‘better’, or if any is ‘valid’, but is only to assess whether there are any important differences in results when using MVN vs ICE-PMM multiple imputation, based on that specific dataset.
3) Multiple-imputation results include a Monte-Carlo (MC) error (due to the finite number of imputation) and results are likely to vary slightly from one set of imputation to another. For example, on the ‘all sample’ dataset, the MC error with 100 imputations for the mean incremental QALYs was around 0.0005 and for incremental costs around 4.9.

[1] Morris, T. P., White, I. R., & Royston, P. (2014). Tuning multiple imputation by predictive mean matching and local residual draws. *BMC medical research methodology*, *14*(1), 75.
